# Current developments of metal- and metalloid-based quinoline compounds as leishmanicidal agents

**DOI:** 10.3389/fchem.2025.1586044

**Published:** 2025-05-30

**Authors:** Edgar Del Carpio, Lino Hernández, Vito Lubes, Francisco Jourdan, Hugo Cerecetto, Gonzalo Scalese, Dinorah Gambino, Angel H. Romero

**Affiliations:** ^1^ Unidad de Química Medicinal, Facultad de Farmacia, Escuela “Dr. Jesús María Bianco”, Universidad Central de Venezuela (UCV), Caracas, Venezuela; ^2^ Departamento de Química, Universidad Simón Bolívar (USB), Caracas, Venezuela; ^3^ Grupo de Química Orgánica Medicinal, Facultad de Ciencias, Universidad de la República, Montevideo, Uruguay; ^4^ Área de Radiofarmacia, Centro de Investigaciones Nucleares, Facultad de Ciencias, Universidad de la República Montevideo, Montevideo, Uruguay; ^5^ Laboratory Redox Biology of Trypanosomes, Institut Pasteur de Montevideo, Montevideo, Uruguay; ^6^ Área Química Inorgánica, Facultad de Química, Universidad de la República, Montevideo, Uruguay

**Keywords:** quinoline, *Leishmania*, ferrocene, N-heterocyclic carbenes, 8-quinolinate

## Abstract

The quinoline moiety represents an important scaffold for the development of leishmanicidal agents. In particular, its hybridization with metal/metalloids has generated highly active compounds that are, in some cases, highly selective against leishmaniasis models. The existing leishmanicidal metal-/metalloid-quinoline compounds are mainly based on the following: (i) coordination compounds based on 8-hydroxyquinolinate; (ii) metallocene derivatives; (iii) *N*-heterocyclic carbene (NHC) complexes featuring a quinoline moiety. This mini-review summarizes the reported cases of leishmanicidal metal and metalloid-based quinoline compounds for each group (i–iii), focusing on the structure-property relationship from *in vitro Leishmania* models and mechanisms of action, *in vivo* experiments, and pharmacokinetic data, if available. This paper aims to describe the state of the art of inorganic medicinal chemistry for the development of selective and potent leishmanicidal agents using the quinoline moiety.

## 1 Introduction

Leishmaniasis, one of the most important neglected tropical diseases, is caused by the protozoan intracellular parasite of *Leishmania* spp (Murray et al., 2005). The disease is present in 98 countries, registering between 0.7 and 1.3 million new cases and approximately 40,000 deaths annually (Kumar, 2021; World Health Organisation, 2023).

Regarding treatment, there are no vaccines, and the therapeutic alternatives are ineffective. Current chemotherapy is primarily based on pentavalent antimonials (e.g., glucantime and pentostam) and pentamidine, which are not approved by the Food and Drug Administration (FDA), and other FDA-approved drugs such as amphotericin B and miltefosine (Aronson et al., 2016; Kumari et al., 2022). However, in general, these commercial drugs present significant side effects (affecting the heart, liver, and kidneys), high cost, low therapeutic efficacy, and prolonged treatment duration (30–60 days) (Nih.gov, 2022). Combination therapies with multiple drugs (Mota et al., 2024; Sundar et al., 2024), development of liposomes and nanoparticles for controlled drug release (Mendes et al., 2020), and repositioning of drugs have been used as emerging therapeutic strategies to improve the efficiency and therapeutic arsenal (Chartlon et al., 2017). Alternatively, the Drugs for Neglected Diseases initiative, (2023) (DNDi) and European and Asian agencies have made great investments in the discovery of new leishmanicidal agents; however, the failure rate has been too high (e.g., from DNDi data, only 20 of 4,200,000 tested) (DNDi, 2023). To develop new effective, selective, and safe leishmanicidal agents, it is necessary to go beyond the classic concept of medicinal chemistry, focusing on key aspects of parasite survival within macrophages (Romero and Delgado, 2024).

Because metal ions play an important role in many biological processes, the use of metal-containing compounds to modulate parasite biological processes has gained relevance as a therapeutic alternative for the design of leishmanicidal agents (Medina-Franco et al., 2022; Gambino and Otero, 2018; Gambino, 2024; Jimenez-Falcao et al., 2024; Hemmert et al., 2025; Tahghighi, 2014). Metal binding can promote the following: (i) DNA interaction; (ii) inhibition of key enzymes; (iii) oxidative stress; (iv) affectation of cell parasite integrity (Rosa et al., 2021; Navarro and Visbal, 2015; Crans and Kostenkova, 2020; Karges et al., 2021; Kostenkova et al., 2022; Fricker et al., 2008; Scalese et al., 2024b; Colotti et al., 2013), among other effects. Because native strains of *Leishmania* have developed arsenic reductase enzymes (Mukhopadhyay et al., 2009), the introduction of reducible elements into leishmanicidal agents can be of great relevance to induce a redox-active pathway within the parasite (Mushtaq et al., 2016; Ouellette et al., 2004). For that reason, the antimonials glucantime or pentostam are among the most representative leishmanicidal agents. Beyond the semimetal Sb(V), Bi(III)/Bi(V) (Duffin et al., 2017; Islam et al., 2014; Fiore-Apuzzo et al., 2021) and transition metals such as Fe, Ru, Re, Mn, Pd, Pt, Rh, Ir, and Au (Ong et al., 2018; Gambino and Otero, 2018; Gambino, 2024; Rosa et al., 2021; Braga et al., 2022) have been used for the development of leishmanicidal agents. Aside from the redox-active element, the choice of the organic ligand plays a key role, and active scaffolds are typically chosen in the design of metal and metalloid-based compounds.

Different organic ligands, including quinolines, salicylaldimines, thiosemicarbazones, and azoles, have been used for the generation of prospective metallodrugs in which the combination enhances the activity and selectivity compared to parent ligands (Gambino and Otero, 2018; Mbaba et al., 2020). In particular, the electron-donor-moiety-substituted quinoline represents a highly convenient scaffold for the design of leishmanicidal agents because i) this structure can be involved in key aspects of *Leishmania* survival during infection process such as parasite mitochondrial dysfunction, accumulation into the phagolysosome, and immunostimulating roles (Romero et al., 2019; Silva et al., 2023; Romero and Delgado, 2024; Yaluf et al., 2025; Avanzo et al., 2025) as well as ii) their multiple feasible synthetic ways (Delgado et al., 2025). The present mini-review aims to provide a general compilation of metal and metalloid-based quinoline compounds as leishmanicidal agents, focusing on biological response against *in vitro* models (promastigote form and/or amastigote form), and limited *in vivo*, structure–activity, and mechanistic aspects. The existing leishmanicidal quinoline-metal compounds are mainly based on the following three structures: (i) coordination compounds based on 8-hydroxyquinolinate; (ii) metallocenes bearing a quinoline moiety; (iii) *N*-heterocyclic carbene (NHC) complexes bearing a quinoline moiety. Herein, to facilitate the analysis, we revised this classification accordingly.

## 2 Metal- and metalloid-based quinoline compounds

### 2.1 Coordination compounds based on 8-hydroxyquinolinates

**FIGURE 1:**
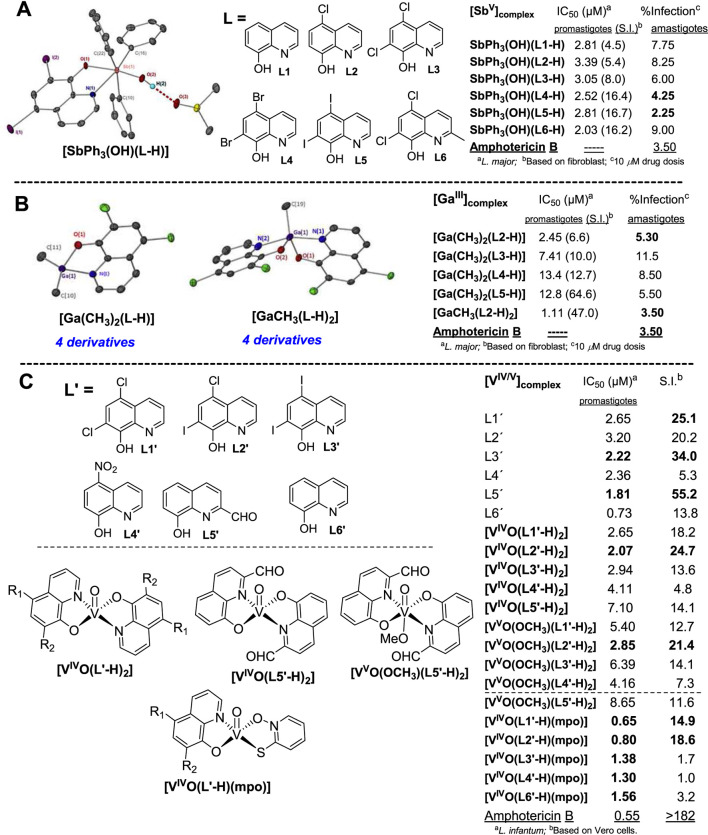
General structure and leishmanicidal response of Sb (V) **(A)**, Ga(III) **(B)**, and vanadium **(C)** complexes featuring 8-hydroxyquinolinates.

Between 2020 and 2021, Duffin prepared a series of Sb(V) and Ga(III) 8-hydroxyquinolinate complexes using different 8-quinolinol substituted ligands (**L1–L6**) to evaluate their activities against promastigotes and amastigotes of *L. major* (Figures 1A,B). Beginning with Sb(V) complexes, six heteroleptic Sb(V) 8-hydroxyquinolinolate compounds, ([SbPh_3_(OH)(L-H)]), were synthesized and characterized (Figure 1A) (Duffin et al., 2021). Single crystal *X-ray* analysis revealed a distorted octahedral geometry with an O-Sb-O angle ranging from 164.3° to 174.0°. Against the promastigote model, the six complexes exhibited similar leishmanicidal responses, displaying IC_50_ values between 2.03 µM and 3.39 μM. Compounds **[SbPh**_**3**_ **(OH)(L4-H)]**, **[SbPh**_**3**_ **(OH)(L5-H)],** and **[SbPh**_**3**_ **(OH)(L6-H)]** showed the highest selectivity indexes (S.I.) (∼16 based on fibroblast cytotoxicity and antipromastigote activity). Against an amastigote model (infection between 15% and 20%) and under a compound treatment (10 μM), the **[SbPh**_**3**_ **(OH)(L4-H)]** and **[SbPh**_**3**_ **(OH)(L5-H)]** reduced the infection to 4.25% and 2.25%, respectively, which was comparable to that obtained under amphotericin B treatment (3.5%). The remaining Sb-quinoline compounds reduced the infection between 7% and 9%.

Regarding Ga(III) complexes, four monomethylgallium (III)([Ga(CH_3_)(L-H)_2_]) and four other dimethylgallium(III) ([Ga(CH_3_)_2_(L-H)]) compounds containing a halogen-substituted 8-hydroxyquinolinate ligand (**L2**, **L3**, **L4,** and **L5**, Figure 1A) were prepared (Duffin et al., 2020). Single-crystal X-ray diffraction showed that the monomethyl complexes were characterized by adopting a five-coordinate trigonal bipyramidal structure, whereas the dimethyl complexes showed a four-coordinate tetrahedral geometry (Figure 1B). **[GaCH**_**3**_ **(L3-H)**_**2**_ **]**, **[GaCH**_**3**_ **(L4-H)**_**2**_ **]**, and **[GaCH**_**3**_ **(L5-H)**_**2**_ **]** complexes were not tested due to their poor solubility. Against the promastigote model, complexes **[Ga(CH**_**3**_ **)**_**2**_ **(L2-H)]** and **[GaCH**_**3**_ **(L2-H)**_**2**_ **]** exhibited the strongest leishmanicidal response with IC_50_ values of 2.45 µM and 1.11 µM, respectively, whereas the remaining compounds displayed IC_50_ values between 7 µM and 13 µM. Against the amastigote model and under 10 µM treatment, complexes **[Ga(CH**_**3**_ **)**_**2**_ **(L2-H)]** and **[GaCH**_**3**_ **(L2-H)**_**2**_ **]** showed superior activity, reducing the infection to 5.3% and 3.5%, respectively, which is comparable with the leishmanicidal response of amphotericin B (3.5%). The **[GaCH**_**3**_ **(L2-H)**_**2**_ **]** complex showed the best S.I. (Scalese et al., 2019), being clearly more selective than Sb(V) analogs (S.I. ∼ 16). These compounds showed good stability at lysosomal pH (Crans and Kostenkova, 2020; Colotti et al., 2013), facilitating accumulation and chemical stability in phagolysosomes (Romero and Delgado, 2022). Overall, the antimonium and gallium coordination improved the leishmanicidal response compared to the parent 8-hydroxyquinolines. Meanwhile, a qualitative structure–activity analysis revealed that polyhalogenated quinolinates bearing heavy halogens such as bromide and iodide led to more active and selective quinolinate-antimonium and -gallium complexes. Regarding the mechanism of action, either Sb(V)- or Ga(III)-8-hydroxyquinolinate complexes promoted the production of ROS in cytosolic macrophages.

Oxidovanadium(IV) and (V) species have demonstrated versatility for drug design due to their diverse therapeutic effects (Del Carpio et al., 2018; Hernández et al., 2022). Gambino´s group synthesized and characterized two groups of V(IV)- and V(V)-8-hydroxyquinolinate complexes, and biologically evaluated them against *L. infantum* promastigotes using ^51^V-NMR, EPR, ESI-MS, and microanalysis. The first group consisted of a series of V(IV) and V(V) bis(8-hydroxyquinolinate) complexes to give five [V^IV^O(L′-H)_2_] and five [V^V^O(OCH_3_)(L′-H)_2_] complexes using 8-hydroxyquinolinate ligands **L1´–L5´** (Figure 1C) (Scalese et al., 2019).

Vanadium complexes (IC_50_ = 2–9 μM, S.I. = 4.8–24.7) were slightly less active and selective than parent 8-quinolinol ligands (IC_50_ = 2–3 μM, S.I. = 5.3–55.2) (Figure 1C). For **L2′**, V(IV)- and V(V)-complexation barely improved both the leishmanicidal response and selectivity of the 8-quinolinol **L2′** from an IC_50_ of 3.2 *µ*M (S.I. = 20.2) to an IC_50_ of 2.07 µM (S.I. = 24.7) and 2.85 *µ*M (S.I. = 21.4) for **[V** ^**IV**^ **O(L2′-H)**_**2**_ **]** and **[V** ^**V**^ **O(OCH**_**3**_ **)(L2′-H)**_**2**_ **]** complexes, respectively. V(IV) complexes were, in general, more active and selective than their V(V) counterparts. Gambino and Otero, (2018) reported the leishmanicidal potential of a second group of heteroleptic V(IV) complexes bearing a 8-hydroxyquinolinate (L1′-L4′ and L6′) and 2-mercaptopyridine *N*-oxide (mpo), [V^IV^O(L′-H)(mpo)] (Figure 1C) (Scalese et al., 2024a). The new vanadium complexes (IC_50_ = 0.65–1.56 µM) were significantly more active than their parent 8-quinolinols (IC_50_ = 0.73–3.20 µM) and V(IV) and V(V) bis(8-quinolinate) complexes (IC_50_∼2–9 µM), although they exhibited a higher toxicity and lower selectivity (S.I. = 1–18.6) than quinolinols (S.I. = 5.3–34) and vanadium-bis(8-hydroxyquinolinate) complexes (S.I. = 4.8–24.7). Among the [V^IV^O(L′-H)(mpo)] complexes, **[V** ^**IV**^ **O(L1′-H)(mpo)]** and **[V** ^**IV**^ **O(L2′-H)(mpo)]** were the most active and selective. Even though incorporating the mpo ligand increases the leishmanicidal response, it compromises the selectivity compared with vanadium-bis(8-hydroxyquinolinate) complexes. Further experiments have shown that vanadium complexes promote late apoptosis/necrosis.

### 2.2 Metallocenes bearing a quinoline moiety

**FIGURE 2:**
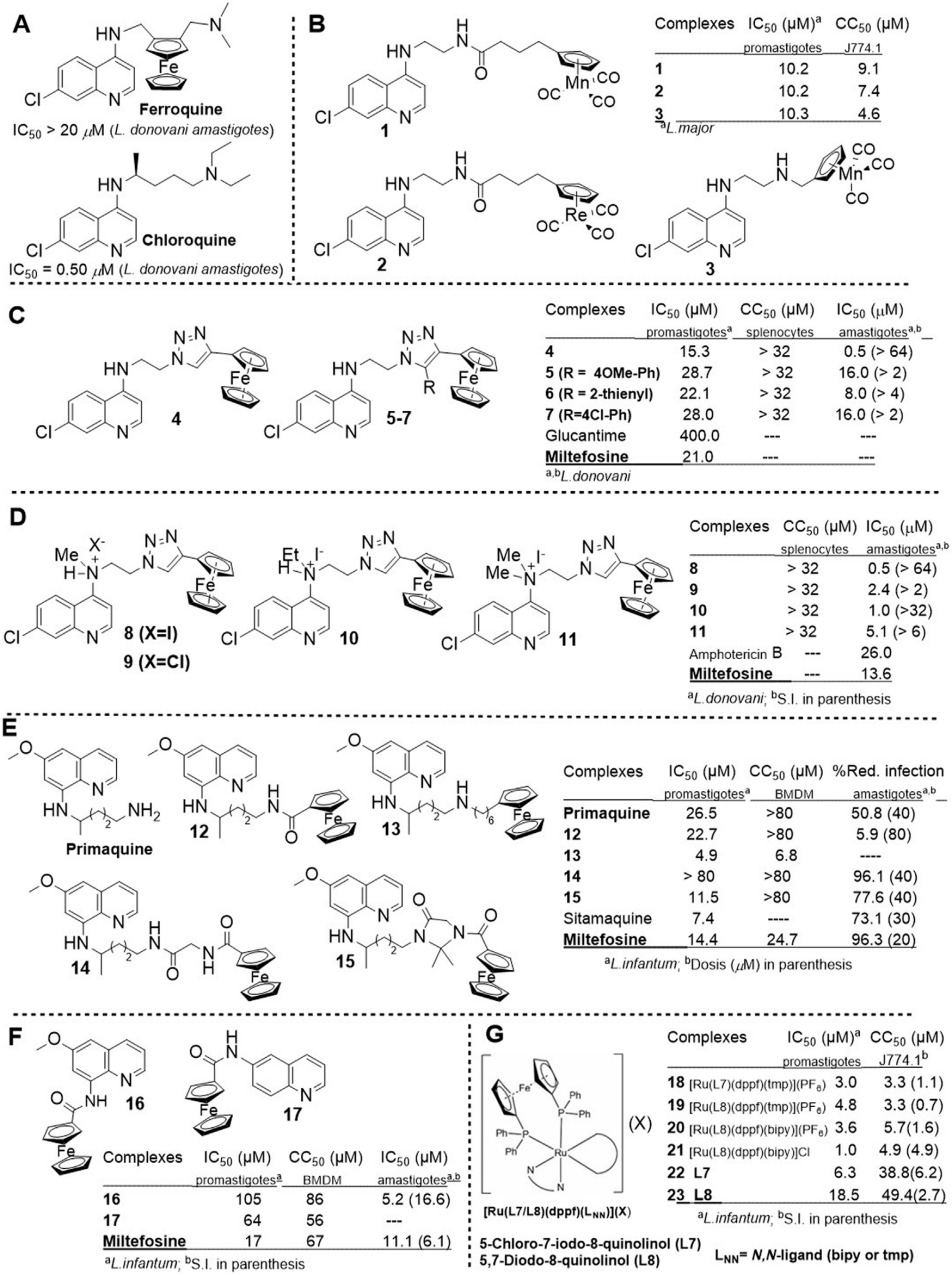
Structure and leishmanicidal activity of quinoline featuring a metallocene moiety **(A–G)**.

Most of these examples are focused on chloroquine analogs bearing a ferrocenyl moiety along the alkylamino lateral chain (Figure 2). Due to the redox properties of the ferrocenyl, that motif favors a high activity against intracellular amastigotes of *Leishmania* spp. (Vale-Costa et al., 2013; Mbaba et al., 2022). The redox activity of the ferrocenyl may also alter parasite metabolism, induce oxidative stress, or interfere with parasite replication or transcription (Kondratskyi et al., 2017). In addition, including the ferrocenyl moiety into the lead structure may favor water solubility, reducing the toxic side effects and improving bioaccumulation (Van Staveren and Metzler-Nolte, 2004).

Our compilation begins with the example reported by Pomel et al. (2015) using ferroquine (Figure 2A). **Ferroquine** is a chloroquine analog bearing a ferrocenyl group along the dialkyldiamino chain. **Ferroquine** was found to be inactive at 20 μM against intracellular amastigotes of *L. donovani*, in contrast with **chloroquine** (IC_50_ = 0.5 μM against *L. donovani* amastigote) (Pomel et al., 2012). Thus, incorporating the ferrocenyl moiety into the middle region of the dialkyldiamino chain appears to be ineffective.

In 2012, Nordlander and co-workers prepared a series of chloroquine analogs bearing a cymantrenyl-, [CpMn(CO)_3_], or cyrhetrenyl-moiety, [CpRe(CO)_3_], at the end of the alkylamino lateral chain (Figure 2B) (Glans et al., 2012). The compounds showed a discrete leishmanicidal response against *L. major* promastigotes, displaying IC_50_ values around 10.2 µM. Another feature that limits the potential of the metallocenes **1–3** is their high toxicities on J774.1 macrophages (CC_50_ = 4.6–9.1 *µ*M), which implies S.I. values lower than 1. Therefore, the cymantrenyl and cyrhetrenyl moieties offer limited potential for enhancing the leishmanicidal activity and selectivity of chloroquine analogs.

Between 2015 and 2016, Adhikari´s group prepared a series of 13 chloroquine analogs featuring a ferrocenyl moiety at the end of the alkylamino lateral chain and a triazolyl moiety to replace the dialkylamino group for evaluation against *Leishmania* spp. (Figure 2C) (Yousuf et al., 2015; Yousuf et al., 2016). Among the 13 derivatives, compounds **4–7** were identified as the most active against *L. donovani* promastigotes (Figure 2C), although displaying a discrete response (IC_50_∼22–28 µM for compounds **5-7** and IC_50_ = 15.3 µM for compound **4**). An extra aryl/heteroaryl ring in the ferrocenyl-quinoline compound did not enhance the leishmanicidal response compared to compound **4**. These drugs had similar leishmanicidal responses to leishmanicidal reference drugs miltefosine (IC_50_ = 21 µM), chloroquine (IC_50_ = 30 µM), and ferroquine (IC_50_ = 20 µM) against a *L. donovani* promastigote. Compound **4** was identified as the most promising compound against the amastigote strain, being able to inhibit the amastigote proliferation in 50% at 0.5 µM without cytotoxicity toward murine splenocytes (at 32 µM treatment), which revealed the high specificity of this type of quinolinic compound toward the amastigote form over the promastigote form. Compounds **5–7** displayed IC_50_ values between 8 µM and 16 µM. Further studies showed that compound **4** promoted: (i) changes in the mitochondrial depolarization potential into promastigotes; (ii) changes promastigote morphology including loss of flagella and appearance of pores in cell membrane; (iii) death via apoptosis; (iv) depletion of GSH; (v) DNA fragmentation in promastigotes; (vi) increase of ROS level; (vii) increase of NO production in infected macrophages model; (viii) increase of the levels of lipid peroxides. Compound 4 acts through an oxidative pathway, initiated by lipid peroxidation, followed by a decrease in protein content and changes in the nature of the lipidic membranes. The latter leads to a loss of mitochondrial membrane potential, leading to apoptosis in *L. donovani* promastigotes. The release of NO in the infected macrophage model could suggest that the compound can also upregulate the innate immune response.

To improve the water solubility of compound **4**, Adhikari´s group prepared a series of four novel flexible and water-soluble ferrocenyl quinolines with *N*-quaternization, **8–11** (Figure 2D) (Mukherjee et al., 2020). Against the intracellular amastigote of *L. donovani*, compound **8** displayed the best leishmanicidal response with an IC_50_ value of 0.50 μM, whereas compounds **9**, **10**, and **11** displayed IC_50_ values of 2.4 µM, 1.0 µM, and 5.1 μM, respectively. Their activities were higher than those of amphotericin B (IC_50_ = 26.0 μM), Glucantime (IC_50_ = 170 μM), miltefosine (IC_50_ = 13.6 μM), and paromomycin (IC_50_ = 8 μM). Compound **8** was evaluated for *in vivo* efficacy and mechanistic assays, demonstrating a significant reduction of parasitemia with a dose-dependent response under oral administration. Further studies showed that the ferrocenyl quinoline **8** stimulated the secretion of Th1 with either oral or intramuscular administration and promoted the expression of key pro-inflammatory cytokines, IL-6, IL-12, TNF-α, and IL-1β for the *in vivo* model. A significant increase in the level of NO in *in vivo* models was found. In addition, compound **8** reduced the expression of key enzymes such as γ-glutamylcysteine synthetase, glutathione synthetase, ornithine decarboxylase, and trypanothione reductase. Additionally, compound **8** showed good pharmacokinetic/pharmacodynamic and oral bioavailability profiles (*C*_*max*_ of 581.8 ng/mL and 287.8 ng/mL, *t*_*1/2*_ of 7.7 h and 15.0 h, AUC_0-inf_ of 14,060.2 g/mL×h and 8077.2 ng/mL×h and a maximum concentration in the liver of 214.7 µg/g and 106.4 μg/g under oral and intramuscular administration, respectively). Compound **8** did not interfere with the expression of phase I and phase II detoxification enzymes in the host liver, making it a good candidate for further preclinical studies.

Beyond the chloroquine scaffold, Vale-Costa prepared a series of thirty primaquine analogs bearing a ferrocene group at the end of the 8-alkylamino lateral chain (Figure 2E) (Vale-Costa et al., 2012) to evaluate against promastigotes and amastigotes of *L. infantum*. Compounds **12–15** showed a good leishmanicidal profile. Against the promastigote model, complexes **13** and **15** exhibited the best antipromastigote response with IC_50_ values of 4.9 µM and 11.5 µM, respectively, which were lower than that found for primaquine (IC_50_ = 26.5 µM) and in the same range as sitamaquine (IC_50_ = 7.4 µM) and miltefosine (IC_50_ = 14.4 µM). Compound **12** showed a discrete response (IC_50_ = 22.7 µM), whereas compound **14** was not active (IC_50_ > 80 µM). Interestingly, the most active, compound **13**, was the most toxic compound (CC_50_ = 6.8 µM), whereas the other three compounds, **12**, **14,** and **15**, and primaquine displayed CC_50_ responses higher than 80 µM. Compounds **12**, **14**, and **15** were evaluated against the amastigote model, finding that only compounds **14** and **15** promoted a significant reduction of infection in infected macrophages by approximately 96% and 77%, respectively, upon a compound treatment of 40 µM. These results demonstrated that the incorporation of the ferrocenyl moiety improved the leishmanicidal response of primaquine, which only showed a reduction of 50.8% under the same conditions. Further assays are needed to evaluate the potential of compounds **14** and **15**.

Another non-chloroquine analog example was described by Madureira and co-workers. They prepared a series of eight 8-*O*, 4-, 3-, 6-, and 8-aminoquinolines connected to a ferrocene group through ester or amide bridges (Quintal et al., 2013) (Figure 2F). Compounds showed a weak leishmanicidal response against promastigotes of *L. infantum*, giving high IC_50_ values (64–269 µM). Only a quinoline–ferrocene derivative, compound **16**, was tested against infected macrophages, displaying a significant reduction of infection with an IC_50_ value of 5.2 µM, which implies an S.I. value of 16.6 that was significantly better than that of the control drug miltefosine (6.1). A comparison with primaquine analogs highlighted that the presence of a longer alkylamino chain between the quinoline core and the ferrocenyl group is essential for generating a more potent and selective agent.

Recently, Gambino (2024) reported the leishmanicidal activity of novel multifunctional Ru(II) ferrocenyl compounds **(18–21)** featuring a single 1,1′-bis (diphenylphosphino) ferrocenyl (dppf), a 8-hydroxyquinolinyl ligand **(22–23)** and a polypyridyl ligand (Figure 2G) (Rivas et al., 2022). Selected 8-hydroxyquinolinyl ligands were 5-chloro-7-iodo-8-hydroxyquinoline **(L7)** and 5,7-diiodo-8-hydroxyquinoline **(L8)**, and selected polypyridyl ligands were 3,4,7,8 tetramethylphenanthroline and 2,2′-bypyridine. These new Ru-Fe compounds enhanced the leishmanicidal effect against the *L. infantum* promastigote by more than 2-fold compared to the parent 8-quinolinols, but they were considerably more toxic on J774.1 macrophages, implying S.I. values between 0.7 and 4.9. Only compound **21**, which features a chloride as counterion, showed an S.I. of 4.9 derived from an IC_50_ of 1.0 µM against promastigote *L. infantum*.

### 2.3 *N*-heterocyclic carbene complexes featuring quinoline moiety

**FIGURE 3:**
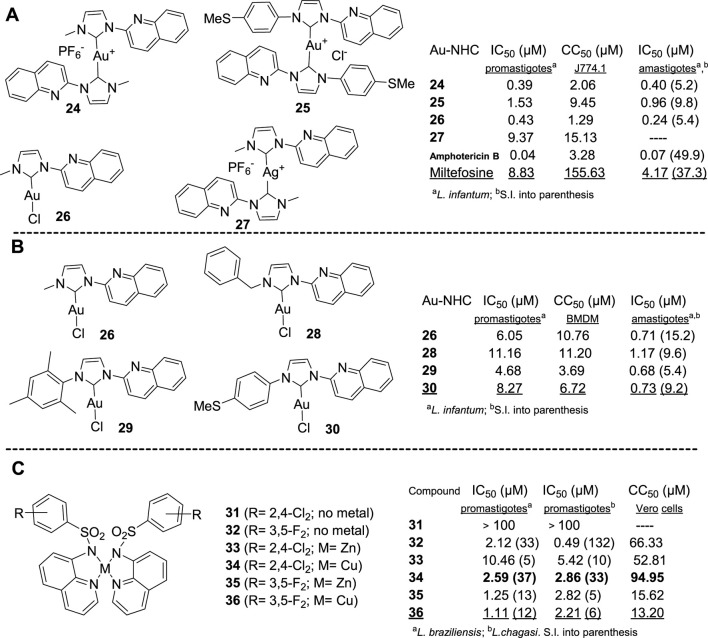
Leishmanicidal response of Au(I)/Ag(I)-NHC complexes bearing a 2-quinoline moiety **24–27 (A)** and **26**, **28–30 (B),** and **(C)** *N*-quinolin-8-yl-arylsulfonamides copper(II) and zinc(II) complexes **(33–36)** from quinoline ligands **31–32**.

Paloque et al. reported in 2015 a series of four organometallic complexes of Au(I) and one of Ag(I) bearing quinoline-functionalized *N*-heterocyclic carbenes (NHC) as ligands (Paloque et al., 2015). In general, the Au(I) NHC complexes **24–26** displayed IC_50_ values against *L. infantum* promastigotes between 0.4 µM and 1.5 µM, whereas the Ag(I) NHC complex **27** showed a higher IC_50_ value of 9.37 µM (Figure 3A). All four Au-NHC compounds exhibited some toxicity against macrophages with CC_50_ values lower than 9 µM. Only the Au(I) NHC complexes **24**, **25**, and **26** were selected for evaluation against the amastigote model due to their higher selectivity indexes of 5.3, 6.2, and 3.0, respectively. Against the amastigote model, the Au(I) NHC complexes **24**, **25**, and **26** displayed IC_50_ values of 0.40 µM, 0.96 µM, and 0.24 µM, respectively, which implies S.I. values of 5.2, 9.8, and 5.4, respectively. These selectivities were lower than those of miltefosine (IC_50_ = 4.17 µM, S.I. = 37.3) and amphotericin (IC_50_ = 0.07 µM; S.I. = 49.9) on amastigotes.

In 2018, Zhang described a series of three new NHC complexes of Au(I) **28–30** bearing a quinoline moiety and a lipophilic chain (e.g., benzyl, 2,4,6-trimethylphenyl, or 4-methylthiophenyl) connected at the imidazolium nitrogens (Zhang et al., 2018) (Figure 3B). Compound **26** was also included in that study. Compounds exhibited a moderate leishmanicidal response against promastigotes with IC_50_ values between 5 µM and 11 µM. These compounds displayed similar IC_50_ concentrations against axenic amastigotes (0.68–1.17 µM). Cytotoxicity on J774.1 macrophages showed that compound **26** was the most promising candidate with an S.I. value of 15.2 from a CC_50_ value of 10.76 µM. Similarly, their activities and selectivities were lower than those of miltefosine (IC_50_ = 0.66 µM, S.I. = 71.8) and amphotericin (IC_50_ = 0.05 µM; S.I. = 24.1) against axenic amastigotes. It seems that a more lipophilic chain at imidazolium, in particular, the 2,4,6-trimethylphenyl one, further compromises the selectivity.

Beyond these three types of structures, an exceptional case of a series of *N*-quinolin-8-yl-arylsulfonamides copper and zinc complexes (Everson da Silva et al., 2010) can be found in the literature. That report described the preparation of eight Zn- or Cu-complexes using four types of *N*-quinolin-8-yl-arylsulfonamides, where the aryl moiety consisted of 8-(*N,N*-dimethylamino) naphthyl and 4-bromo-, 3,4-dichloro-, 3,5-difluorophenyl. The compounds, ligands, and complexes (some selected cases **31–36** are shown in Figure 3C) were evaluated against promastigote *L. braziliensis* and *L. chagasi* promastigotes, finding that only the copper complexation of the *N*-quinolin-8-yl-(2′,4′-dichlorophenyl) sulfonamide provided a potent and selective agent **34** giving IC_50_ values of 2.59 µM and 2.86 µM against these *Leishmania* species, respectively. That complexation significantly enhanced the leishmanicidal effect compared with the parent quinoline **31**. Compound **34** displayed an IC_50_ value of 0.35 µM against intracellular amastigotes of *L. braziliensis*. The zinc and copper complexation of *N*-quinolin-8-yl-(3′,5′-difluorophenyl) sulfonamide and *N*-quinolin-8-yl-(4′-bromophenyl) sulfonamide did not improve the leishmanicidal effect and increased the cytotoxicity on Vero cells, generating less selective compounds than the parent quinolines. Moreover, the complexation of *N*-quinolin-8-(8′-*N,N*-dimethylaminonaphthyl) sulfonamide yielded inactive compounds (IC_50_ > 100 µM).

## 3 Conclusion

This mini-review provided a general overview of the state of the art in developing leishmanicidal metal and metalloid compounds based on quinolines. As described, the leishmanicidal quinoline-metal compounds are mainly based on: (i) Sb(V), Ga(III), or V(IV/V) bearing 8-hydroxyquinolinates as a ligand; (ii) chloroquine or primaquine analogs bearing a ferrocenyl moiety at the alkylamino chain; (iii) quinolines bearing an Au-NHC moiety. From the first group, three key remarks were extracted: (i) dihalogenated-quinolinols generated the most selective and active complexes, (ii) Ga(III) generated the most selective and least toxic agent compared to the Sb(V) and V(IV/V) analogs, and (iii) this type of complex is able to promote ROS production. From the second group, it should be noted that: (i) the ferrocenyl moiety generated more active and selective agents than group VII-metals (Mn, Re) cymantrenyl and cyrhetrenyl organometallic derivatives; (ii) the incorporation of the ferrocenyl moiety at the terminal position of the dialkyldiamino chain in chloroquine or primaquine analogs improves selectivity more than its incorporation at the middle region; (iii) more promising agents were found by using active quinoline (chloroquine or primaquine) than a non-active quinoline platform; (iv) cations of ferrocenyl quinolines are highly convenient to generate water-soluble compounds with appropriate physicochemical properties; (v) the ferrocenyl quinolines promoted a specific mechanism based on oxidative stress-inducing morphological changes and early apoptosis and promoting immunostimulation in an infected animal model; (vi) a ferrocene–quinoline compound, compound **8**, was identified as promising agent by its curative effect and parasitemia reduction in *in vivo* models of visceral leishmaniasis and by its excellent pharmacokinetic/pharmacodynamics and oral bioavailability. Further investigations of the metal-NHC-quinolines are needed to optimize the structures for lowering toxicity on the host cells, which compromises selectivity.

Considering the potential of the ferrocenyl motif-derived compounds to generate active and selective antileishmanial agents, metals and metalloids containing quinoline ferrocenyl derivatives are promising hit compounds for the development of new agents for the treatment of leishmaniasis. In addition, further preclinical investigations are needed to evaluate the potential of other promising compounds (e.g., [Ga(CH_3_)(L2-H)_2_], [Ga(CH_3_)_2_(L5-H)], and compounds **10**, **14**, and **34**) as leishmanicidals. Collectively, the reported data underscore the need for further exploration of metal and metalloid quinoline derivatives as promising candidates in the development of novel antileishmanial agents.
